# Longitudinal Trajectories of Food Insecurity in Childhood and Their Associations With Mental Health and Functioning in Adolescence

**DOI:** 10.1001/jamanetworkopen.2021.40085

**Published:** 2021-12-20

**Authors:** Vincent Paquin, Gina Muckle, Despina Bolanis, Yohann Courtemanche, Natalie Castellanos-Ryan, Michel Boivin, Richard Tremblay, Sylvana Côté, Marie-Claude Geoffroy

**Affiliations:** 1 McGill Group for Suicide Studies, Department of Psychiatry, Douglas Mental Health University Institute, Montreal, Québec, Canada; 2 School of Psychology, Laval University, Québec City, Québec, Canada; 3 Centre Hospitalier Universitaire de Québec, Laval University Research Center, Québec City, Québec, Canada; 4 Department of Educational and Counselling Psychology, McGill University, Montreal, Québec, Canada; 5 Department of Psychology, University of Montreal, Montreal, Québec, Canada; 6 Centre Hospitalier Universitaire Sainte-Justine Research Centre, Montreal, Québec, Canada; 7 Department of Social and Preventive Medicine, University of Montreal, Montreal, Québec, Canada; 8 INSERM 1219 Bordeaux Population Health, Bordeaux, France

## Abstract

**Importance:**

Household food insecurity has been associated with mental health problems in children independently of family income and other confounders. It is unclear whether food insecurity during childhood is also associated with mental health and functioning during adolescence.

**Objective:**

To evaluate longitudinal trajectories of household food insecurity during the first 13 years of life, characteristics associated with these trajectories, and the associations of the trajectories with externalizing, internalizing, substance use, and social adjustment problems at 15 years of age.

**Design, Setting, and Participants:**

This cohort study included participants from the Québec Longitudinal Study of Child Development, a cohort of 2120 children born in Québec, Canada, in 1997 and 1998 and followed up annually or biannually from 5 months to 15 years of age (1998-2013). Data were analyzed from November 2020 to October 2021.

**Exposures:**

When children were aged 1.5, 4, 8, 10, 12, and 13 years, mothers reported whether a family member experienced hunger because the family had run out of food or money to buy food in the past 12 months.

**Main Outcomes and Measures:**

Group-based trajectory modeling was used to identify differential exposure to food insecurity from 1.5 to 13 years according to logit functions of age. At 15 years of age, adolescents completed validated questionnaires assessing externalizing, internalizing, substance use, and social adjustment problems. Associations between trajectories of food insecurity and outcomes were assessed using linear regressions.

**Results:**

A total of 2032 individuals were included in the analyses of trajectories of food insecurity (1026 [50.5%] male) and 1441 in the analysis of the association with outcomes at 15 years of age (752 [52.2%] female). Two trajectories of food insecurity were identified between 1.5 and 13 years of age: high risk (73 children [3.6%]) and low risk (1959 children [96.4%]). At 5 months, the high-risk and low-risk groups differed in household characteristics including income insufficiency (58 [80.6%] vs 405 [21.0%]), single parenthood (21 [29.2%] vs 135 [6.9%]), and parental history of depression (mothers: 30 [43.5%] vs 411 [21.7%]; fathers: 12 [32.4%] vs 209 [13.5%]). The high-risk trajectory from 1.5 to 13 years of age was associated with cannabis use (β, 0.47; 95% CI, 0.12-0.81), peer bullying (β, 0.43; 95% CI, 0.08-0.77), and dropout potential (β, 0.38; 95% CI, 0.03-0.68) at 15 years of age after adjustment for sex, household income insufficiency, and parental mental health.

**Conclusions and Relevance:**

In this cohort study, few children experienced a persistent high risk of food insecurity, which was associated with psychosocial problems later in adolescence after adjustment for confounders including low income. Early identification of risk for food insecurity may guide the delivery of tailored interventions to improve functioning in adolescence.

## Key Points

**Question:**

Are there associations between trajectories of household food insecurity during childhood and mental health and functioning during adolescence?

**Findings:**

In this cohort study of 2032 infants who were followed up from 5 months to 15 years of age, 2 trajectories of household food insecurity were identified between 1.5 and 13 years of age: low risk (96.4%) and high risk (3.6%). High risk for food insecurity was significantly associated with cannabis use, peer bullying, and school dropout potential at 15 years of age after adjustment for sex, household income sufficiency, and parental mental health.

**Meaning:**

The findings suggest that food insecurity can be identified early in life and that its recurrence is associated with poorer functioning during adolescence.

## Teaser

This cohort study of Canadian infants followed up from age 5 months to 15 years examines the trajectories of food insecurity during childhood and the association of food insecurity with mental health and social functioning outcomes at 15 years of age.

## Introduction

From 2017 through 2018, 13% of Canadian households had food insecurity in the past year (with a similar prevalence in the US), which means they were “at times, unable to acquire adequate food for one or more household members because they had insufficient money and other resources for food.”^1,2^ The prevalence of food insecurity increased during the COVID-19 pandemic in 2020.^3,4,5^ Presence of children, lower income, lower educational attainment, and migrant status were associated with household food insecurity.^5^

Children are particularly at risk for the consequences of food insecurity because they are rapidly developing.^6,7,8^ Household food insecurity during childhood has been associated with negative outcomes independently of low income and other key confounders. Prospective associations of food insecurity with obesity, academic difficulties, internalizing and externalizing problems, and poorer social skills have been reported in population-based cohorts.^9,10,11^ For example, in the Québec Longitudinal Study of Child Development (QLSCD), researchers found an association of food insecurity at 1.5 and 4.5 years of age with maternal reports of hyperactivity or inattention problems persisting from 4.5 to 8 years of age.^11^ Less research has examined outcomes of food insecurity during adolescence,^7^ another key developmental period in which most mental health disorders emerge.^12^ Of the few studies examining outcomes during adolescence, most have been cross-sectional (from which the temporal precedence cannot be established)^7,8^ or limited to specific outcomes such as misconduct^13^ or depression.^14^

Longitudinal profiles of childhood food insecurity may be differentially associated with developmental outcomes during adolescence. Similar to poverty,^15,16,17,18,19^ food insecurity can be transient or recurrent, and recurrent food insecurity may be a household marker of risk for poorer health outcomes.^10,20^ However, most studies have typically relied on few measures of food insecurity over intervals of 2 to 4 years that have often been aggregated into a single indicator of any past food insecurity during childhood or adolescence.^7,8^ Tracking trajectories of food insecurity and their correlates from the first years of life to the transition into adolescence may guide the tailoring of interventions for at-risk households. Using the QLSCD, a large Canadian birth cohort prospectively followed up for 15 years, we aimed to (1) identify longitudinal trajectories of food insecurity during childhood and the sociodemographic characteristics associated with these trajectories and (2) examine the associations of the trajectories with a range of mental health and social functioning outcomes during adolescence.

## Methods

### Participants

Participants for this cohort study were identified from the QLSCD (conducted by the Institut de la Statistique du Québec), a population-based birth cohort of 2120 children born in Québec, Canada, in 1997 and 1998 and followed up annually or biannually to present.^21^ The QLSCD was approved by the ethical committees of the Institut de la Statistique du Québec and Centre Hospitalier Universitaire Sainte-Justine Hospital Research Centre. Informed written consent, assent, or both were obtained for each data collection. This study followed the Strengthening the Reporting of Observational Studies in Epidemiology (STROBE) reporting guideline.^22^

We used information on household characteristics reported by both parents at 5 months and 2.5 years of age (1998 and 2000, respectively), food insecurity reported by mothers from 1.5 to 13 years of age (1999-2011), and mental health and functioning outcomes reported by adolescents at 15 years of age (2013). Analyses were conducted between November 2020 and October 2021.

### Household Characteristics

At 5 months of age, we measured household income insufficiency (ie, spending more than 20% of the annual income for basic needs, in addition to the average proportion spent by households of similar size and regional population density^23^). For parents, we also measured educational attainment, migrant status (born in Canada: yes or no), and history of antisocial behavior during adolescence (based on 5 items adapted from the Diagnostic Interview Schedule^24^). At 2.5 years of age, lifetime history of depression in parents was assessed with 16 items from the Diagnostic Interview Schedule.^25^

### Household Food Insecurity Exposure

Food insecurity was reported by mothers when children were 1.5, 4, 8, 10, 12, and 13 years of age in response to the following question: “In the past 12 months, has a member of your family ever experienced being hungry because the family had run out of food or money to buy food?” We coded responses as 0 (no) or 1 (yes; grouping “occasionally,” “certain months,” “every month,” or “regularly” because prevalence in the category was low).

### Mental Health and Functioning Outcomes

All outcomes were self-reported by adolescents at 15 years of age. Four broad domains were investigated: externalizing problems (ie, attention-deficit/ hyperactivity disorder [ADHD] symptoms, opposition or defiance, and conduct), internalizing problems (ie, depression, social anxiety, and symptoms of generalized anxiety disorder), substance use (ie, frequency of alcohol and cannabis use), and social adjustment problems (ie, peer bullying, dropout potential). Higher scores indicated more severe problems.

Adolescents completed the Mental Health and Social Inadaptation Assessment for Adolescents^26^ to assess the frequency in the past 12 months of the following problems (1 indicated never; 2, sometimes, and 3, often): ADHD symptoms (16 items), oppositional or defiance problems (9 items), conduct problems (16 items), depression (8 items), generalized anxiety (9 items), and social anxiety symptoms (7 items). These scales are presented in the eMethods in the Supplement.

Substance use in the past year was self-reported using 2 items from the Detection of Alcohol and Drug Problems in Adolescents, a screening grid for problematic alcohol and drug use validated in adolescent populations.^27^ Frequency of use of alcohol and cannabis (“cannabis, marijuana, pot, hashish”) was assessed by responses to the following question: “During the past 12 months, how often did you use…?” Responses ranged from 1 (“I didn't”) to 7 (“every day”).

Peer bullying was assessed with 6 items adapted from the Social Experience Questionnaire^28^ documenting overt (eg, “Someone pushed, shoved, hit, or kicked me”) and relational (eg, “Someone didn't let me be part of his or her group when I wanted to”) forms of bullying. Responses ranged from 1 (“never”) to 4 (“very often [more than once a week on average]”).

Dropout potential was assessed using the Dropout Prediction Index.^29^ This 9-item questionnaire combines measures of school performance (eg, “What are your average grades in math this school year?”), grade failure (eg, “Have you ever repeated a grade?”), and student engagement (eg, “How important is it to you to get good grades?”) to provide a continuous score of susceptibility for dropping out of high school.^29^

### Statistical Analysis

We applied group-based trajectory modeling^30^ in Mplus, version 7.4 (Muthén & Muthén), to identify differential exposure to food insecurity from 1.5 to 13 years of age according to logit functions. Models of 1- to 4-trajectory groups were estimated using full information maximum likelihood based on the maximum available sample; participants with no food insecurity data across time points were excluded. The best-fitting model was selected using the bayesian information criterion, Lo-Mendell-Rubin likelihood ratio test, and entropy. We compared early-life characteristics associated with food insecurity trajectories using *t* tests and χ^2^ tests.

We examined associations between trajectories of food insecurity and outcomes using linear regressions in R, version 3.6.1 (R Foundation for Statistical Computing). All outcomes were normally distributed except for conduct problems, cannabis use frequency, and bullying (eTable 1 in the Supplement). Outcomes were standardized (mean, 0; SD, 1) so that the regression coefficient (β) represents the proportion of 1 SD of difference in outcomes associated with 1 trajectory compared with the reference trajectory. Effect sizes were interpreted as small (β = 0.2), medium (β = 0.5), and large (β = 0.8).^31^ Directed acyclic graphs ^32,33^ were used to select potential confounding factors (eMethods and eFigures 1 and 2 in the Supplement). We controlled hierarchically for sex, income sufficiency, and parental mental health. Significance was set at *P* < .05 (2-tailed). Trajectory-by-sex interactions were not significant at *P* < .05; therefore, we present results with male and female individuals combined. To account for multiple testing, we present results before and after Bonferroni corrections were applied within domains of outcomes. To explore associations of food insecurity with severe or clinical outcomes and to confirm findings with positively skewed outcomes, we applied logistic regressions to dichotomized outcomes (cutoffs given in eTable 1 in the Supplement).

Characteristics of participants who completed at least 1 questionnaire at 15 years of age (ie, included participants) were compared with those who did not complete a questionnaire (ie, excluded participants) using *t* tests and χ^2^ tests. To adjust for selective attrition at 15 years of age, we conducted sensitivity analyses with inverse probability weights, representing participants’ probabilities of being included conditionally on attrition-associated early life characteristics. Among included participants, data were missing for less than 1% of participants on outcomes and less than 3% of participants on covariables except for paternal depression (13%) and antisocial problems (18%). To minimize further data loss, we imputed missing data using multiple imputations with chained equations with the mice package in R.^34^ For each variable to impute, approximately 20 predictors were procedurally selected from a large set of variables^21^ based on a criterion of *r* > 0.15 with the imputed variable^34,35^; 40 data sets were generated.

## Results

### Trajectories of Food Insecurity From 1.5 to 13 Years of Age and Associated Early-Life Characteristics

**Figure 1.**
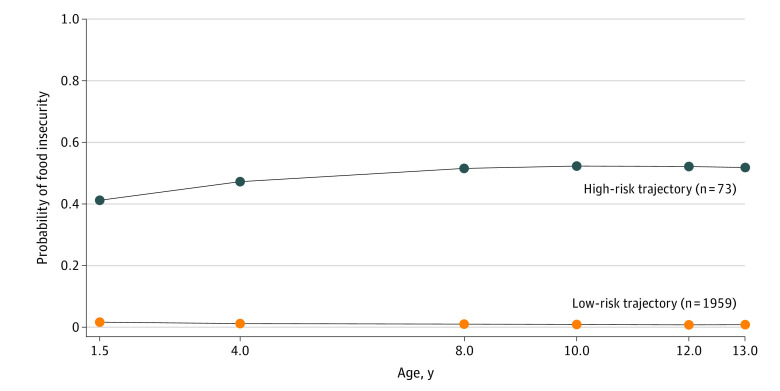
Two-Group Model Trajectories of Food Insecurity From 1.5 to 13 Years of Age Data were compiled from the final master file of the Québec Longitudinal Study of Child Development (1998-2013), Gouvernement du Québec, Institut de la Statistique du Québec. Fit indexes for the models were as follows: 1-group model (bayesian information criterion [BIC], 2379.221; entropy, not applicable; Lo-Mendell-Rubin likelihood ratio test [LMR-LRT], not applicable); 2-group model (BIC, 2066.058; entropy, 0.907; LMR-LRT, *P* < .001); 3-group model (BIC, 2087.434; entropy, 0.782; LMR-LRT, *P* = .20); 4-group model (BIC, 2108.74; entropy, 0.924; LMR-LRT, *P* = .32).

Of 2120 participants at inception of the study, 1288 (60.8%) provided information on food insecurity on at least 4 of the 6 assessments and 2032 (95.8%; 1026 [50.5%] male) provided information on food insecurity on at least 1 occasion and were included in this analysis (Table). The 2-group model best fitted food insecurity trajectories (Figure 1 and eTable 2 in the Supplement). Most children (1959 [96.4%]) had very low (approximately 1%) probability of food insecurity over time (low-risk trajectory). The other children (73 [3.6%]) had a stable, high-risk trajectory of recurrent food insecurity, with approximately 50% probability of experiencing food insecurity at each age. In the high-risk group, all children were exposed to food insecurity at least once, and 56 (76.7%) were exposed at least twice (eTable 3 in the Supplement). In the low-risk group, most children (1857 [94.8%]) were never exposed to food insecurity, 102 (5.2%) were exposed once, and none were exposed more than once.

**Table.** Early-Life Characteristics of Participants by Trajectories of Food Insecurity From 1.5 to 13 Years of Age^a^

Characteristic	Participants, No. (%)		*P* value
	Low-risk trajectory (n = 1959)	High-risk trajectory (n = 73)	
Sex			
Female	967 (49.4)	39 (53.4)	.57
Male	992 (50.6)	34 (46.6)	
Insufficient household income^b^	405 (21.0)	58 (80.6)	<.001
Siblings			
0	832 (42.5)	21 (28.8)	<.001
1	790 (40.3)	25 (34.2)	
≥2	337 (17.2)	27 (37.0)	
Single parent	135 (6.9)	21 (29.2)	<.001
Maternal educational attainment			
No high school diploma	484 (24.7)	39 (53.4)	<.001
High school diploma or higher	1474 (75.3)	34 (46.6)	
Paternal educational attainment			
No high school diploma	508 (28.1)	30 (58.8)	<.001
High school diploma or higher	1300 (71.9)	21 (41.2)	
Born in Canada			
Mother	1755 (89.6)	63 (86.3)	.47
Father	1619 (88.9)	42 (80.8)	.11
History of depression^c^			
Maternal	411 (21.7)	30 (43.5)	<.001
Paternal	209 (13.5)	12 (32.4)	.002
Antisocial behaviors, mean (SD)^d^			
Maternal	0.81 (0.93)	1.02 (1.12)	.15
Paternal	0.67 (0.95)	0.92 (1.15)	.14

^a^ Data were compiled from the final master file of the Québec Longitudinal Study of Child Development (1998-2013), Gouvernement du Québec, Institut de la Statistique du Québec. Variables were measured when the child was 5 months of age unless otherwise indicated.

^b^ Cutoff for household income insufficiency was spending more than 20% of the annual income for basic needs, in addition to the average proportion spent by households of similar size and regional population density.

^c^ Parental lifetime depression history was assessed at 29 months using 16 items adapted from the depression section of the Diagnostic Interview Schedule.^24^

^d^ Parental antisocial behaviors during adolescence were assessed using retrospective items on 5 conduct problems (eg, having been in >1 fight that they started; having stolen >1 time).^24^ Scores ranged from 0 to 5.

As shown in the Table, in the high-risk food insecurity group (n = 73) vs the low-risk group (n = 1959), there were substantially more families with insufficient income (58 [80.6%] vs 405 [21.0%]), low level of education (mothers: 39 [53.4%] vs 484 [24.7%]; fathers: 30 [58.8%] vs 508 [28.1%]), and single parents (21 [29.2%] vs 135 [6.9%]). Children at high risk for food insecurity were more likely to have more siblings (≥2 siblings: 27 [37.0%] vs 337 [17.2%]), and their parents were more likely to have a history of depression (mothers: 30 [43.5%] vs 411 [21.7%]; fathers: 12 [32.4%] vs 209 [13.5%]).

### Associations of Food Insecurity Trajectories With Outcomes at 15 Years of Age

A total of 1441 participants (752 [52.2%] female) were followed up to 15 years of age (eTable 4 in the Supplement), and 679 were lost to follow-up. Adolescents lost to follow-up (eTable 4 in the Supplement) were more likely to be male, live in households with lower socioeconomic status, and live with a single parent; their mothers were more likely to be born outside Canada and to have more symptoms of depression.

**Figure 2.**
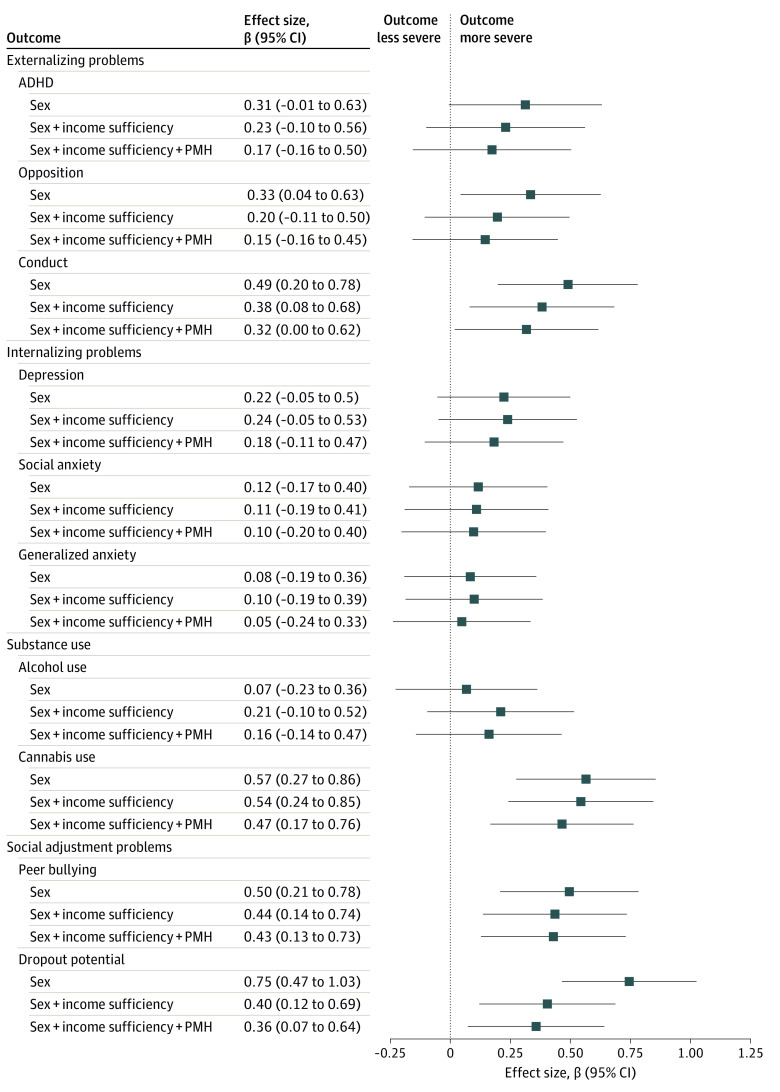
Associations Between High-risk Trajectory of Food Insecurity From 1.5 to 13 Years of Age and Mental Health and Functioning at 15 Years of Age Data were compiled from the final master file of the Québec Longitudinal Study of Child Development (1998-2013), Gouvernement du Québec, Institut de la Statistique du Québec. Standardized coefficients (β) of linear regressions were pooled over 40 multiply imputed data sets (n = 1441). The reference category was the low-risk trajectory of food insecurity. Covariables were entered hierarchically as follows: (1) sex, (2) income sufficiency at 5 months, and (3) parental mental health (PMH) (history of depression measured at 29 months and antisocial behaviors during adolescence measured at 5 months). ADHD indicates attention-deficit/hyperactivity disorder symptoms.

In fully adjusted models, the high-risk trajectory of food insecurity was associated with higher levels of conduct problems, cannabis use, peer bullying, and dropout potential compared with the low-risk trajectory; effect sizes were small to moderate (Figure 2 and eTable 5 in the Supplement). After Bonferroni corrections, associations with cannabis use (β, 0.47; 95% CI, 0.12-0.81), peer bullying (β, 0.43; 95% CI, 0.08-0.77), and dropout potential (β, 0.38; 95% CI, 0.03-0.68) remained significant, whereas the association with conduct problems did not (β, 0.32; 95% CI, −0.05 to 0.68). In the attrition-adjusted sensitivity analysis, the models yielded wider 95% CIs but a similar pattern of findings (eFigure 4 in the Supplement). In logistic regression analyses, we found significant associations of the high-risk trajectory with frequent cannabis use (monthly or more often: odds ratio [OR], 2.77; 95% CI, 1.32-5.83), severe bullying (≥90th percentile: OR, 2.54; 95% CI, 1.18-5.50), and dropout risk (scores ≥0.40: OR, 1.98; 95% CI, 1.04-3.75), but not with other outcomes (eFigure 5 in the Supplement).

## Discussion

In this population-based cohort study from Québec, Canada, we found that 3.6% of children had stable, high risk for recurrent food insecurity, whereas 96.4% of children had consistently low risk over time. Characteristics at birth, including household income insufficiency, greater number of siblings, and poorer parental mental health, were associated with the high-risk trajectory of food insecurity. High risk for food insecurity during childhood was associated with higher levels of cannabis use, peer bullying, and school dropout potential at 15 years of age. These associations were present for both continuous and dichotomized (severe) outcome levels, and they were robust to adjustment for income insufficiency and parental mental health.

In the general population of Québec, Canada, from 2011 to 2012, the prevalence of household food insecurity was 8%, and large households, single-parent households, lower income level, and lower educational attainment were associated with greater risk of food insecurity.^36^ In a representative cohort of 11 958 US children,^37^ 6.5% were food insecure in both kindergarten and first grade (ie, persistent during 2010-2011). As expected, given the shorter follow-up period, this estimate was above the prevalence of high (recurrent) risk for food insecurity reported in the present study (ie, 3.6%). Prevalence of recurrent food insecurity during childhood has been reported elsewhere but in more socioeconomically disadvantaged cohorts,^10,20,38^ limiting comparisons with our study.

Associations of food insecurity with externalizing problems beyond familial characteristics, including parental income, depression, and education,^10,11,13^ are supported by previous research. After adjustment for sex only in the present study, the high-risk trajectory of food insecurity was associated with externalizing problems at 15 years of age, with small effect sizes for ADHD and opposition and a moderate effect size for conduct problems. For ADHD and opposition, the associations did not persist after adjusting for income insufficiency and parental mental health, whereas the association with conduct problems was no longer significant after Bonferroni corrections. This lack of an association with externalizing problems contradicts a previous QLSCD study^11^ showing that food insecurity at 1.5 to 4.5 years of age was associated with maternal reports of hyperactivity and inattention problems at 4.5 to 8 years of age. To our knowledge, other studies of food insecurity and ADHD in youths have been cross-sectional,^8^ and most have relied on maternal reports of ADHD. Maternal ratings of ADHD may be influenced by parental distress,^39,40,41^ which may be associated with food insecurity,^42,43^ thus possibly inflating observed associations between food insecurity and ADHD. In the current investigation, the use of adolescent ratings probably mitigated the association of parental distress with outcome measurements.

We found no association between the high-risk trajectory of food insecurity and internalizing problems. A recent systematic review^7^ supports an association of food insecurity with depression and anxiety during adolescence, but most of the included studies were cross-sectional. An exception was the Fragile Families and Child Wellbeing Study (2626 US children),^42^ a longitudinal study that reported an association between maternal reports of food insecurity at 5 years of age and adolescent reports of symptoms of depression and anxiety at 15 years of age. However, socioeconomically disadvantaged households were overrepresented in that cohort, which likely enriched the exposure group with more severe food insecurity.

In the present study, we found longitudinal associations of childhood food insecurity with cannabis use, peer bullying, and dropout potential during adolescence (all medium effect sizes in the fully adjusted models) that were consistent with findings of prior cross-sectional research. Food insecurity was cross-sectionally associated with odds of substance use disorder in a national US survey of youths aged 13 to 17 years.^44^ In a representative survey of 2063 US adolescents aged 12 to 16 years, household food insecurity was associated with difficulties getting along with peers and suspension from school,^45^ which may reflect greater propensity for bullying and dropout.

Familial dynamics related to food insecurity may explain the observed associations with cannabis use, bullying, and dropout potential. Either as a proxy or a risk factor, childhood food insecurity has been shown to be associated with interpersonal adversity in the familial and social environments.^46,47,48^ For example, household food insecurity may be associated with increased parental stress, leading to more intimate partner violence^46,47^ and child maltreatment^48^ in affected families. In turn, by affecting how a child learns to interact with others and to cope with stress, this interpersonal adversity during childhood may be associated with subsequently higher levels of cannabis use,^49^ bullying,^50^ and academic difficulties^51^ during adolescence. However, despite these psychosocial correlates, food insecurity was not associated with internalizing and externalizing behaviors in the present study. To yield more tangible differences in mental health, food insecurity may require interaction with other forms of adversity or genetic factors.^52,53,54^

Our study showed that a high risk for food insecurity was likely to persist throughout childhood and to be associated with complex problems during adolescence. Although supplying food to families in precarious situations is a vital service and may play a role in alleviating some of this stress,^6^ we suggest that children at high risk for food insecurity receive broader psychosocial services (eg, targeting the familial and social environments) to help them fulfill their academic and social potentials.

### Strengths and Limitations

This study has strengths. We used 6 repeated measures of food insecurity to capture longitudinal risk profiles in a population-based sample of children. Multidomain assessment of mental health and functioning in adolescence provided a comprehensive picture of outcomes prospectively associated with food insecurity risk, bringing some insight into potential underlying mechanisms and service needs.

This study also has limitations. We captured food insecurity in households and not directly among children. Our measure focused on food insufficiency but not on its quality or diversity, which are important aspects of food security.^6^ More in-depth questionnaires such as the 18-item US Department of Agriculture Food Security Module, which assesses parent and child food sufficiency, quality, and diversity, are considered the gold standards.^6,7^ Still, previous research supports the validity of single-item food insecurity screeners such as ours with respect to health, well-being, and development.^7,8^ Other studies have examined hunger among youths, which represents severe food insecurity, and found associations with depression during adolescence and early adulthood.^55^ As for outcomes, we relied on self-reports from adolescents, who may have underestimated their externalizing problems.^56^ However, having different informants for food insecurity (mothers) and outcomes has the benefit of minimizing shared variance from single informants. Another limitation of our study stems from attrition. Although trajectories were derived from nearly the entire sample, there was an underrepresentation of participants from socioeconomically disadvantaged households at 15 years of age. Attrition-adjusted models produced similar effect sizes, reducing the concern for attrition bias, but the large 95% CIs indicate the need for replication in more representative samples. In addition, we did not evaluate age-specific effects of food insecurity. Age at exposure to food insecurity (eg, infancy vs adolescence) may also be associated with outcomes.^52,57^ Because of sparse, overlapping exposure data, we were unable to explore this in the cohort. Our assessment of food insecurity began at 1.5 years of age; therefore, the prevalence and contribution to outcomes of earlier (eg, prenatal) onset of food insecurity could not be examined.

## Conclusions

In this cohort study, we found 2 trajectories of household food insecurity in children aged 1.5 to 13 years: a high-risk trajectory of exposure associated with socioeconomic precarity and poorer parental mental health and a low-risk trajectory associated with more socioeconomically favorable characteristics in the first years of life. High risk for food insecurity persisted throughout childhood and was associated with greater cannabis use, bullying by peers, and dropout potential during adolescence. These prospective associations support a role for early interventions in food-insecure households.

## Notes

Supplement.
eMethods.
**eFigure 1.** Directed Acyclic Graph of Food Insecurity, Mental Health and Related Factors **eFigure 2.** Minimal Set of Control Variables Identified Via a Directed Acyclic Graph **eFigure 3.** Bivariate Correlations Among Trajectory of Food Insecurity, Sex, Parental Characteristics, and Outcomes in Adolescence **eFigure 4.** Associations Between High-Risk Trajectory of Food Insecurity and Mental Health and Functioning in Adolescence, with Inverse Probability Weights **eFigure 5.** Associations Between High-Risk Trajectory of Food Insecurity and Mental Health and Functioning Problems in Adolescence (Logistic Regressions) **eTable 1.** Descriptive Statistics of Mental Health and Functioning Measures at Age 15 **eTable 2.** Two-Group Model Estimates for Food Insecurity from Age 1.5 to 13 Years **eTable 3.** Prevalence of and Cumulative Exposure to Food Insecurity Between Ages 1.5–13 Years According to Group Trajectories **eTable 4.** Early Life Characteristics of Participants According to Attrition at Age 15 **eTable 5.** Associations Between High-Risk Trajectory of Food Insecurity (1.5–13 years) and Mental Health and Functioning in Adolescence (15 years)
eReferences
