# Supplementary material for: Longitudinal Trajectories of Food Insecurity in Childhood and Their Associations With Mental Health and Functioning in Adolescence
Source: JAMA Netw Open. 2021 Dec 20;4(12):e2140085. doi: 10.1001/jamanetworkopen.2021.40085 (PMC8689386; doi:10.1001/jamanetworkopen.2021.40085)
Supplement: Supplement. — eMethods. eFigure 1. Directed Acyclic Graph of Food Insecurity, Mental Health and Related Factors eFigure 2. Minimal Set of Control Variables Identified Via a Directed Acyclic Graph eFigure 3. Bivariate Correlations Among Trajectory of Food Insecurity, Sex, Parental Characteristics, and Outcomes in Adolescence eFigure 4. Associations Between High-Risk Trajectory of Food Insecurity and Mental Health and Functioning in Adolescence, with Inverse Probability Weights eFigure 5. Associations Between High-Risk Trajectory of Food Insecurity and Mental Health and Functioning Problems in Adolescence (Logistic Regressions) eTable 1. Descriptive Statistics of Mental Health and Functioning Measures at Age 15 eTable 2. Two-Group Model Estimates for Food Insecurity from Age 1.5 to 13 Years eTable 3. Prevalence of and Cumulative Exposure to Food Insecurity Between Ages 1.5–13 Years According to Group Trajectories eTable 4. Early Life Characteristics of Participants According to Attrition at Age 15 eTable 5. Associations Between High-Risk Trajectory of Food Insecurity (1.5–13 years) and Mental Health and Functioning in Adolescence (15 years) eReferences [file jamanetwopen-e2140085-s001.pdf]

Supplementary Online Content

Paquin V, Muckle G, Bolanis D, et al. Longitudinal trajectories of food insecurity in childhood and their associations with mental health and functioning in adolescence. *JAMA Netw Open*. 2021;4(12):e 2140085. doi:10.1001/jamanetworkopen.2021.40085

eMethods.
eFigure 1. Directed Acyclic Graph of Food Insecurity, Mental Health and Related Factors
eFigure 2. Minimal Set of Control Variables Identified Via a Directed Acyclic Graph
eFigure 3. Bivariate Correlations Among Trajectory of Food Insecurity, Sex, Parental Characteristics, and Outcomes in Adolescence
eFigure 4. Associations Between High-Risk Trajectory of Food Insecurity and Mental Health and Functioning in Adolescence, with Inverse Probability Weights
eFigure 5. Associations Between High-Risk Trajectory of Food Insecurity and Mental Health and Functioning Problems in Adolescence (Logistic Regressions)
eTable 1. Descriptive Statistics of Mental Health and Functioning Measures at Age 15
eTable 2. Two-Group Model Estimates for Food Insecurity from Age 1.5 to 13 Years
eTable 3. Prevalence of and Cumulative Exposure to Food Insecurity Between Ages 1.5–13 Years According to Group Trajectories
eTable 4. Early Life Characteristics of Participants According to Attrition at Age 15
eTable 5. Associations Between High-Risk Trajectory of Food Insecurity (1.5–13 years) and Mental Health and Functioning in Adolescence (15 years)
eReferences.

This supplementary material has been provided by the authors to give readers additional information about their work.

© 2021 Paquin V et al. *JAMA Network Open*.

eMethods

Subscales from the Mental Health and Social Inadaptation Assessment

All scales were shown to have good to excellent internal reliability and good convergent and discriminant validity in the current sample.[1] Response options for all items below were: 1=never, 2=sometimes, and 3=often.

Attention Deficit and Hyperactivity

1. I was impulsive (reacted quickly without thinking).
2. I said things before thinking them through.
3. I did or said things without stopping to think.
4. I had difficulty waiting for my turn in games or group activities.
5. I often blurted out the answer to a question that hadn't yet been completely asked.
6. I got into trouble because I did things without thinking.
7. I felt very restless, I was constantly on the move.
8. I often stood up in class or in other situations where I was supposed to remain seated.
9. I often had trouble staying calm during games or leisure activities.
10. I moved my hands and feet, I wriggled in my chair.
11. I was inattentive, I had difficulty paying attention to what someone was saying or doing.
12. I completed all of my tasks or homework, I was able to stay focused.
13. I had trouble keeping my mind on what I was doing for more than a few minutes.
14. I forgot what I was supposed to be doing or what I had planned to do.
15. I avoided doing things where I needed to pay attention for a long time.
16. I made a lot of mistakes because it was hard for me to do things carefully.

Oppositional/Defiant

1. I refused to do what my parents or my teacher were telling me to do.
2. I felt sorry after doing something wrong.
3. I had temper tantrums.
4. I lost my temper easily.
5. I was mean to certain people.
6. I got even with someone by trying to hurt them.
7. I got even with someone by telling lies about them.
8. I blamed someone else when I got caught doing something wrong.
9. I did some things just to annoy people or make them angry.

Conduct

1. I cheated in order to succeed at school.
2. I cheated in order to make some money.
3. I cheated in order to win a competition.
4. I told lies in order to get things or favours from others.
5. I told lies in order to get out of doing things I was supposed to do.
6. I stole money or objects from home.
7. I stole money or objects from school or from stores.
8. I used a weapon in order to steal.
9. I entered a house, a building or a car without permission in order to steal.
10. I broke down a door or a window in order to get into a place and take something.
11. I stayed out at night much later than I was allowed to.
12. I stayed out all night without my parents' permission.
13. I ran away from home.
14. I skipped school without reason (cut class).
15. I deliberately started a fire.
16. I deliberately destroyed someone else's property.

Depression

1. Nothing was fun for me, I wasn't interested in anything.
2. I felt sad and unhappy.
3. I lacked energy or felt tired.
4. I lost interest in things I usually like.
5. I felt I couldn't do anything well.
6. I felt I wasn't as good-looking or as smart as other people.
7. Doing even little things made me feel really tired.

8. I had trouble thinking clearly.

Generalized Anxiety

1. I was too fearful or nervous.
2. I had worries that interfered with my everyday life.
3. I worried about my past behaviour.
4. I worried about my school work.
5. I worried about my own health.
6. I worried about my loved ones (family, friends).
7. I worried about my relationships with my friends (i.e. making and keeping friends).
8. I was concerned about my appearance or weight.
9. I found it difficult to control the worry.

Social Anxiety

1. I feared or tried to avoid situations that involved a lot of people
2. I feared or tried to avoid situations that involved meeting new people
3. I feared or tried to avoid situations that involved doing things in front of an audience
4. I feared or tried to avoid situations that involved speaking in class.
5. I feared or tried to avoid situations that involved reading out loud in front of others
6. I disliked being placed in social situations that drew attention to me.
7. Although I was with people that I trust, I feared social situations that drew attention to me
8. I blushed or trembled when faced with social situations that I fear.

Directed Acyclic Graph for Covariable Selection

The 5 confounding variables controlled for in our analyses (i.e., income sufficiency, maternal/paternal depression history, and maternal/paternal antisocial behaviors in adolescence) were selected *a priori* using a directed acyclic graph (DAG). DAGs are visual tools that illustrate assumptions of causal associations between variables.[2] Traditional methods of covariable selection may inadvertently introduce conditional associations between exposure and outcome, creating bias instead of reducing it. Established analytical rules for DAGs allow the analyst to identify a set of control variables that will minimize bias, based on the directionality of associations between exposure, covariables, and outcome.[2]

A DAG shows confounding ("backdoor") pathways which are non-causal paths between an exposure and an outcome. Using a validated algorithm,[2,3] the DAG allows us to identify the minimal set(s) of confounders that need to be controlled to block all backdoor pathways. Multiple potential confounders may lie along a single backdoor pathway, but controlling one is sufficient to block the overall pathway along which they lie – assuming there is no other pathway connecting these confounders to the exposure and outcome. See Suttorp et al.[4] for further explanations and an example.

Potential confounding factors were identified from the literature[5–10] for their documented associations with food insecurity and/or adolescent mental health. We built the DAG using the "ggdag" package in R,[3] which applies the aforementioned analytical rules to identify the minimal set of confounding factors to adjust for. Below are the causal assumptions that were entered in the graph (see eFigures 1–2 for the DAG and its set of control variables).

Each bullet point below addresses one downstream variable (on the left-hand side of the tilde [~], in bold) and describes all the upstream variables (on the right-hand side of the tilde) that are assumed to be directly (causally) associated with it. These direct causal associations are assembled in the DAG to visually represent both direct and indirect pathways of causations between all variables.

- **MENTAL HEALTH (15 y.o.)** ~ FOOD INSECURITY (Childhood) + Parental Mental Illness (Lifetime) + Parental Education (At Birth) + Childhood Adverse Experiences + Child Ethnicity + Genetic Risk + In Utero and Perinatal Factors + Maternal Age at Birth + Monoparental + Sex + Prenatal Substance Use,
- **FOOD INSECURITY (Childhood)** ~ Parental Income (At Birth) + Parental Mental Illness (Lifetime) + Number of Siblings + Monoparental,
- **In Utero and Perinatal Factors** ~ Parental Mental Illness (Lifetime) + Parental Education (At Birth) + Genetic Risk + Maternal Age at Birth + Monoparental + Prenatal Substance Use + Sex,
- **Prenatal Substance Use** ~ Parental Mental Illness (Lifetime) + Parental Education (At Birth),
- **Maternal Age at Birth** ~ Parental Mental Illness (Lifetime) + Parental Education (At Birth),
- **Monoparental** ~ Parental Mental Illness (Lifetime),
- **Child Ethnicity** ~ Parental Migration,
- **Number of Siblings** ~ Parental Education (At Birth) + Parental Mental Illness (Lifetime) + Parental Migration + Parental Income (At Birth) + Maternal Age at Birth + Monoparental,
- **Parental Migration** ~ Parental Education (At Birth),
- **Parental Income (At Birth)** ~ Parental Education (At Birth) + Parental Mental Illness (Lifetime) + Parental Migrant Status + Maternal Age at Birth + Monoparental,
- **Parental Education (At Birth)** ~ Genetic Risk + Parental Mental Illness (Lifetime),
- **Childhood Adverse Experiences** ~ Parental Mental Illness (Lifetime) + Parental Income (At Birth) + Number of Siblings + FOOD INSECURITY (Childhood) + Monoparental
- **Parental Mental Illness (Lifetime)** ~ Genetic Risk + Parental Migration

eFigure 1. Directed Acyclic Graph of Food Insecurity, Mental Health and Related Factors

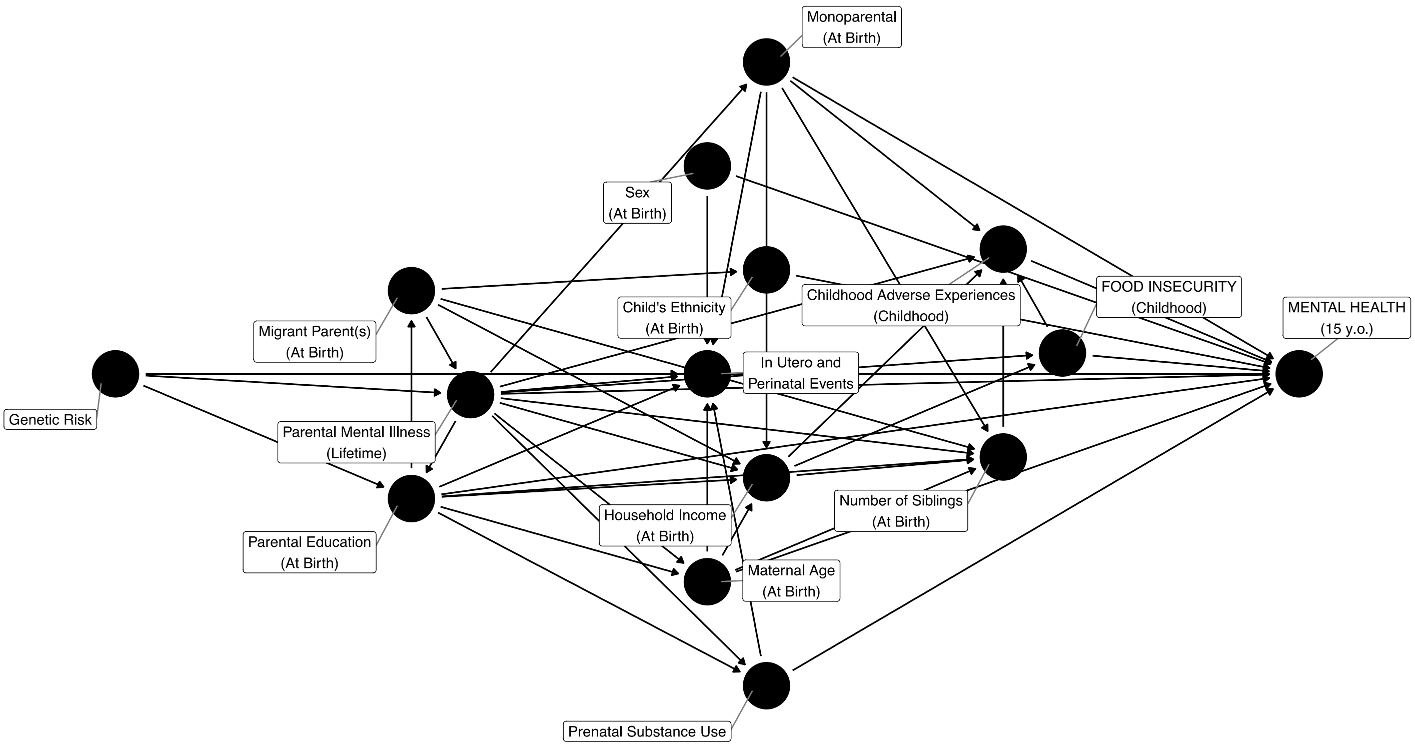

Arrows indicate putative causal associations. Factors are ordered in a semi-chronological order from left to right. Household Income: household income sufficiency based on gross annual income, household size and urbanicity.

© 2021 Paquin V et al. *JAMA Network Open*.

eFigure 2. Minimal Set of Control Variables Identified Via a Directed Acyclic Graph

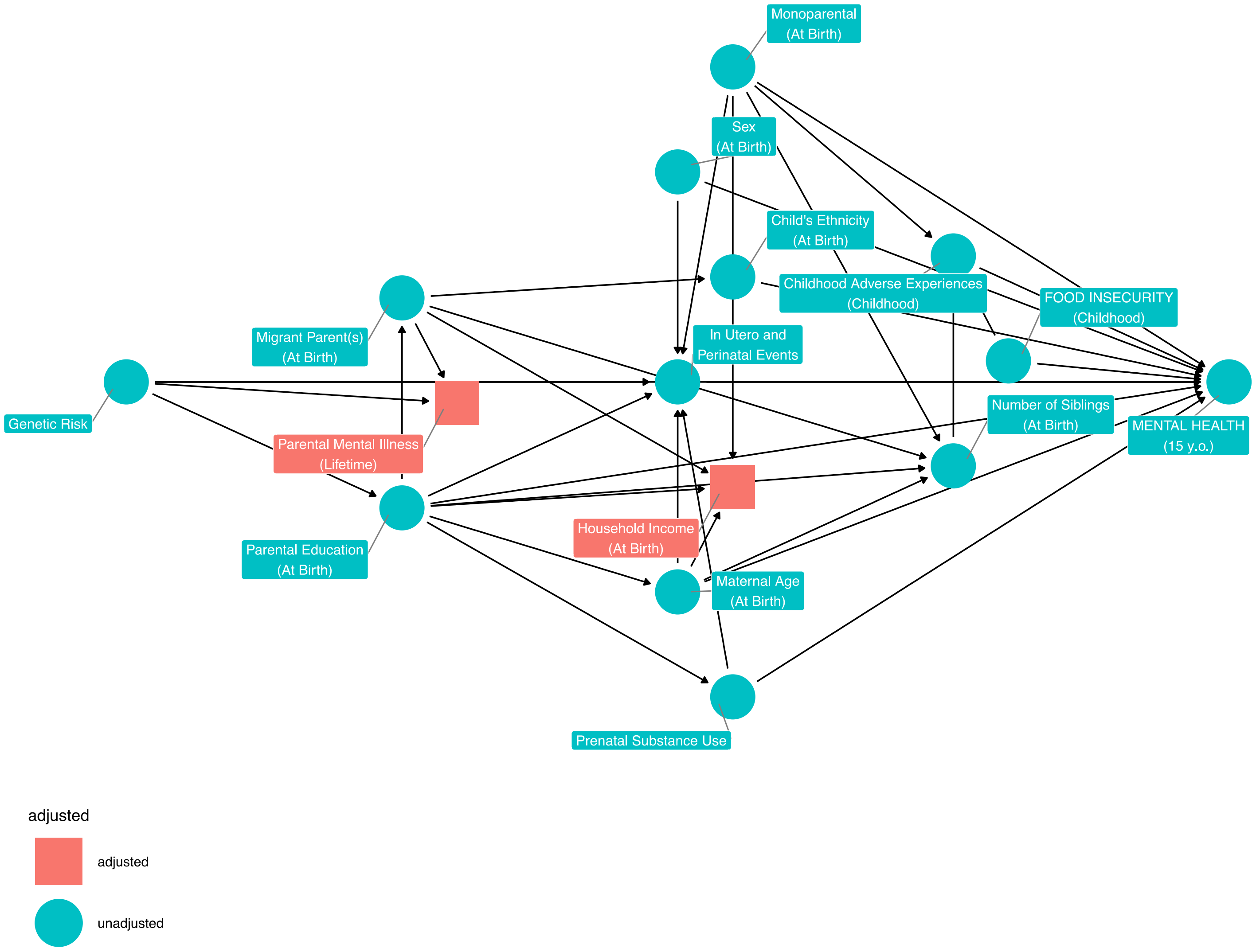

Parental mental illness (lifetime history) and household income sufficiency (accounting for household size and urbanicity) constitute the minimal set of control variables required to block pathways of confounding effects on the outcome (adolescent mental health). No other minimal sets were identified from the graph.

eFigure 3. Bivariate Correlations Among Trajectory of Food Insecurity, Sex, Parental Characteristics, and Outcomes in Adolescence

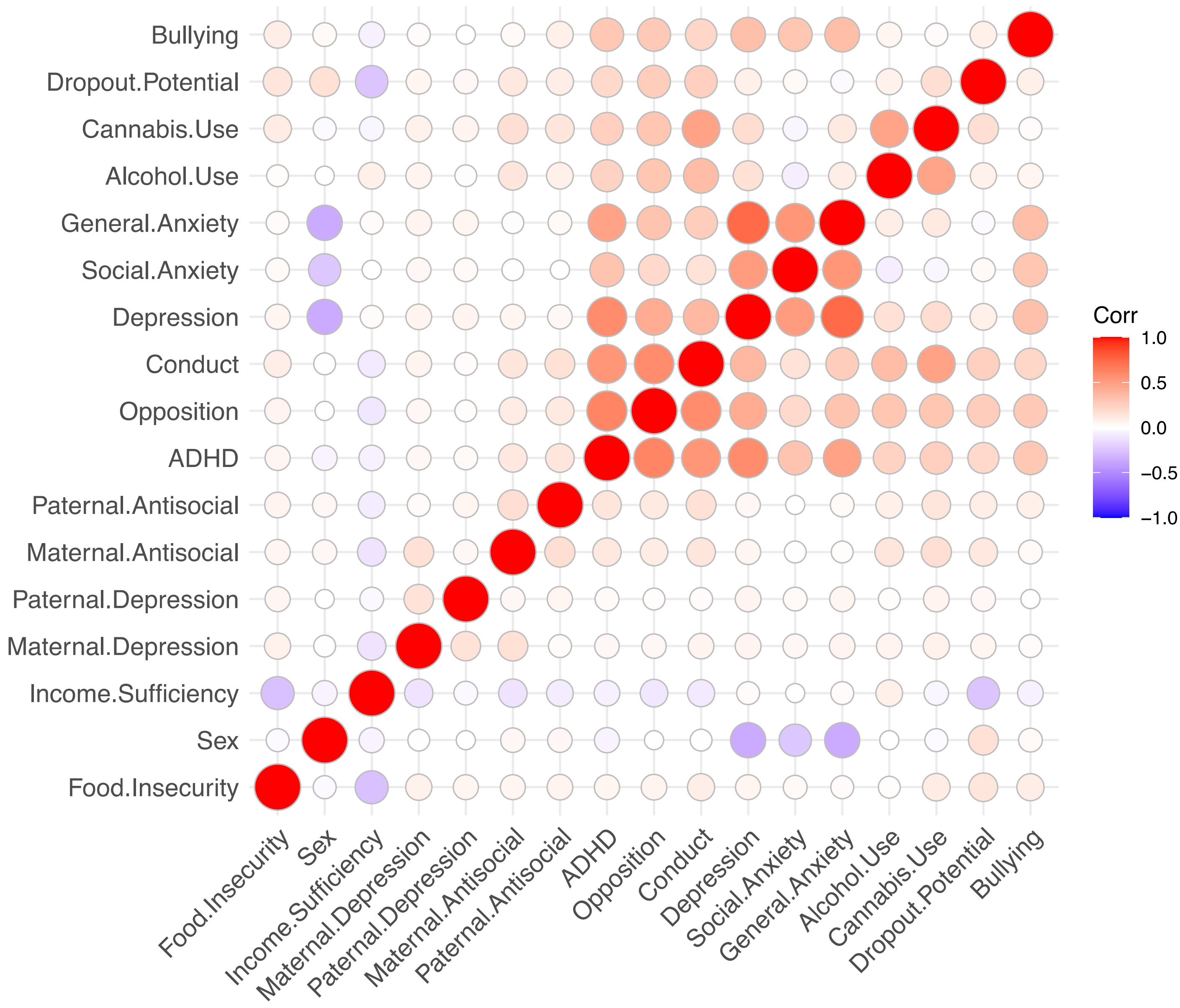

Data were compiled from the final master file of the Québec Longitudinal Study of Child Development (1998–2013), ©Gouvernement du Québec, Institut de la Statistique du Québec. Variables are labeled from bottom to top (y-axis) and left to right (x-axis). Food insecurity trajectory: dichotomized as high risk vs. low risk for food insecurity (between age 1.5 and 13 years). Variables from "Sex" to "Maternal/Parental.Antisocial" (i.e., antisocial behaviors in adolescence) were measured at age 5 months, except "Maternal/Paternal Depression" (i.e., history of depression, measured at 29 months). Variables from "ADHD" (i.e., attention deficit and hyperactivity) to "Bullying" were measured at age 15 years. Corr: Pearson correlations among pairwise complete observations. Income Sufficiency: household income sufficiency (dichotomous) based on gross annual income, household size, and urbanicity.

eFigure 4. Associations Between High-Risk Trajectory of Food Insecurity and Mental Health and Functioning in Adolescence, with Inverse Probability Weights

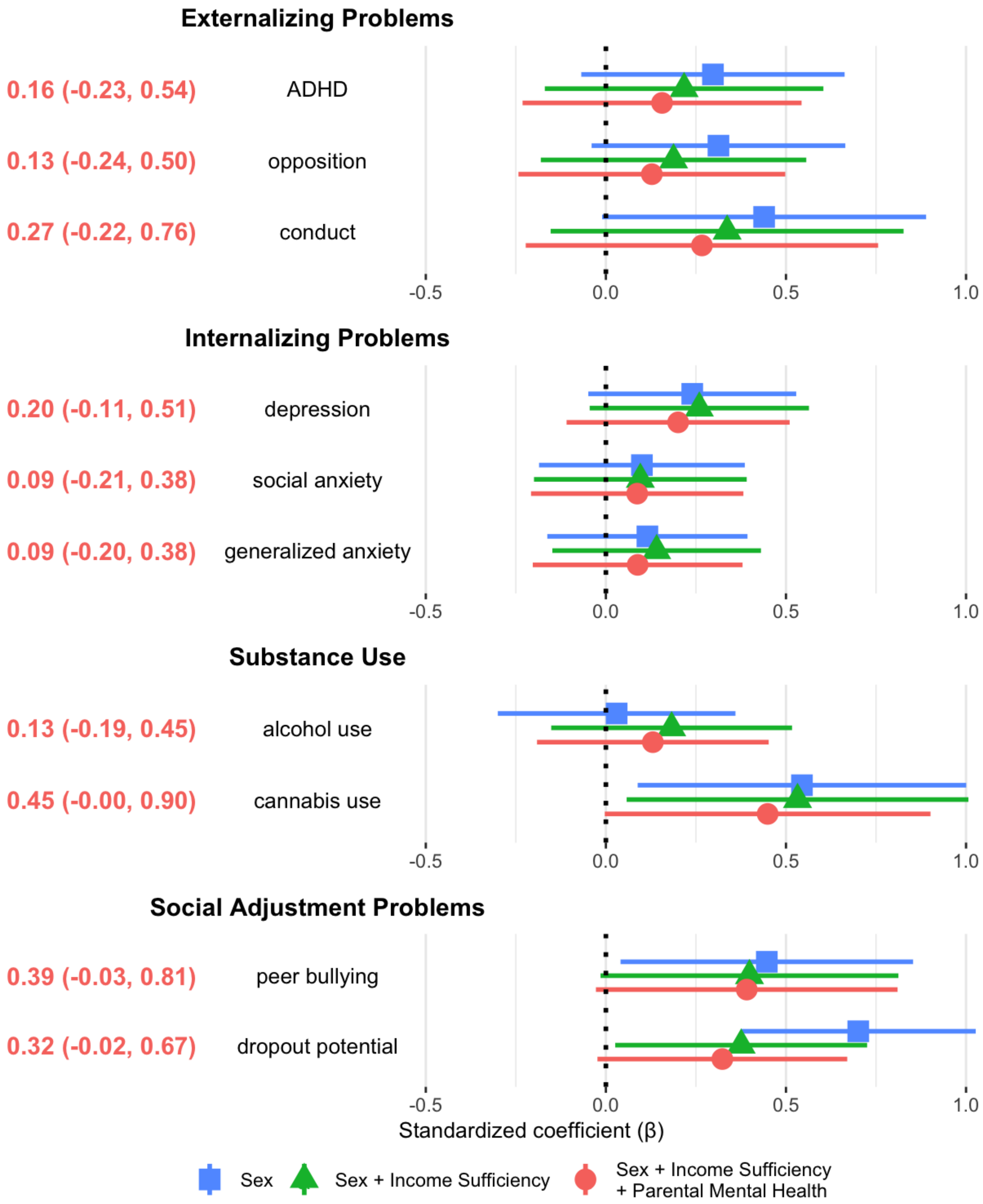

Data were compiled from the final master file of the Québec Longitudinal Study of Child Development (1998–2013), ©Gouvernement du Québec, Institut de la Statistique du Québec. Standardized coefficients (β) of linear regressions were pooled over 40 multiply imputed datasets (n=1441). Inverse probability weights were conditioned on sex, socioeconomic status, maternal migrant status and maternal depressive symptoms according to inclusion in the analytic sample. Left-hand side: β in the fully adjusted models. Covariables were entered hierarchically as follows: (1) sex, (2) income sufficiency at 5 months, and (3) parental mental health. ADHD indicates attention deficit and hyperactivity disorder symptoms.

eFigure 5. Associations Between High-Risk Trajectory of Food Insecurity and Mental Health and Functioning Problems in Adolescence (Logistic Regressions).

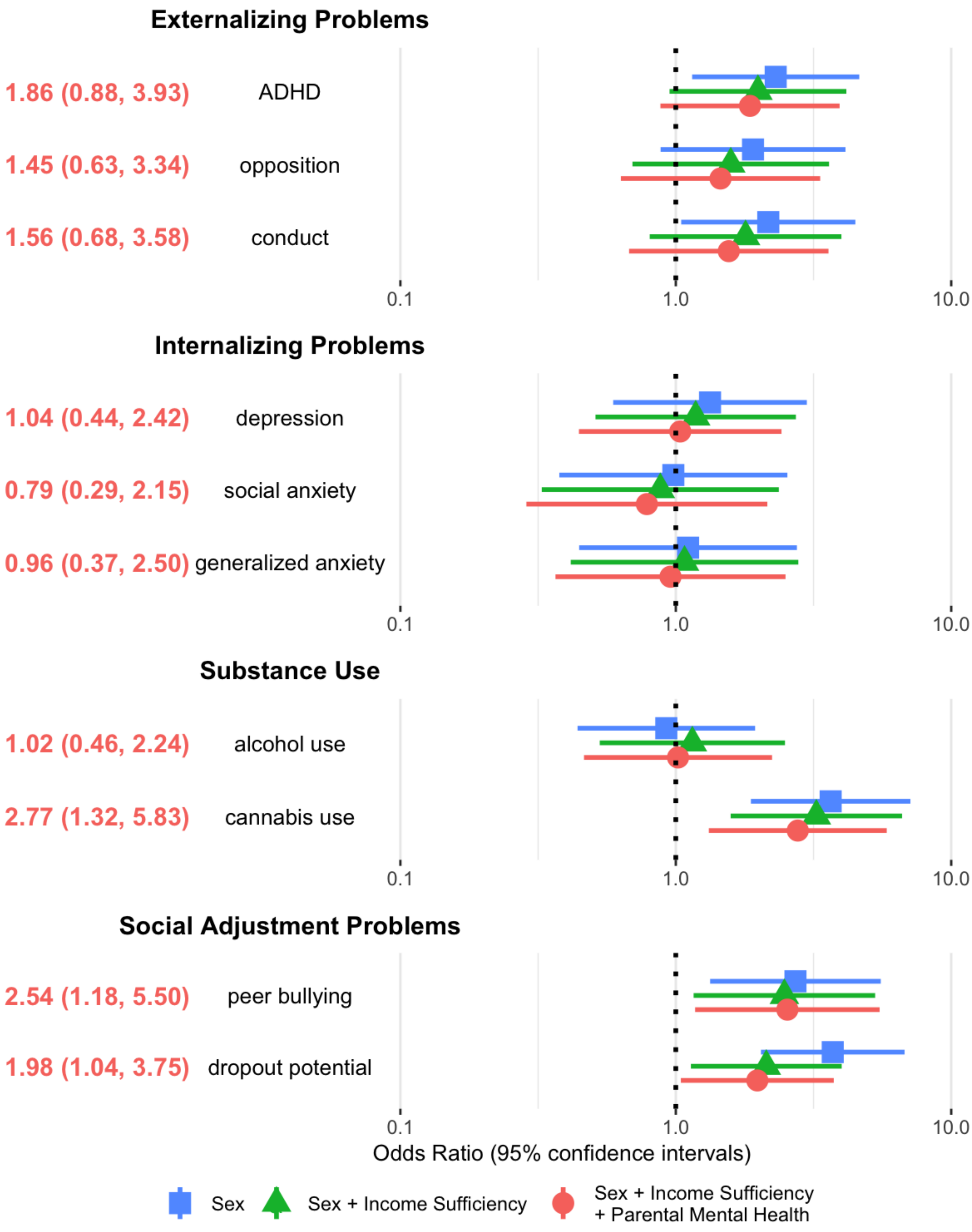

Data were compiled from the final master file of the Québec Longitudinal Study of Child Development (1998–2013), ©Gouvernement du Québec, Institut de la Statistique du Québec. Associations are between the high-risk trajectory of food insecurity (relative to low-risk trajectory) and odds of severe outcomes (definition for internalizing, externalizing, and victimization problems: ≥90th percentiles;[11] for alcohol and cannabis: monthly use or more; for dropout potential: scores ≥ 0.40[12]). Logistic regressions were pooled over 40 multiply imputed datasets (n=1441). Left-hand side: odds ratio in the fully adjusted models. Covariables were entered hierarchically as follows: (1) sex, (2) income sufficiency at 5 months, and (3) parental mental health. ADHD indicates attention deficit and hyperactivity disorder symptoms.

eTable 1. Descriptive Statistics of Mental Health and Functioning Measures at Age 15

Outcome	Mean	Standard deviation	Median	Interquartile range	Cutoff for severity
Externalizing Problems					
ADHD	2.90	1.67	2.78	2.36	5.14
Opposition	2.35	1.39	2.22	2.22	4.44
Conduct	0.65	0.90	0.31	0.94	1.56
Internalizing Problems					
Depression	3.48	2.24	3.13	3.12	6.88
Social anxiety	2.40	2.17	1.88	3.12	5.63
Generalized anxiety	4.09	2.17	3.89	3.34	7.22
Substance Use					
Alcohol use	2.41	1.34	3.00	2.00	4.00
Cannabis use	1.64	1.36	1.00	0.00	4.00
Social Adjustment Problems					
Peer bullying	1.22	1.45	0.60	1.70	2.81
Dropout potential	0.27	0.27	0.15	0.33	0.40

Data were compiled from the final master file of the Québec Longitudinal Study of Child Development (1998-2013), ©Gouvernement du Québec, Institut de la Statistique du Québec. Descriptive statistics were pooled over 40 multiply imputed datasets (n=1441). Scores for externalizing problems, internalizing problems and peer bullying were rescaled to be expressed on a scale from 0 to 10.[1] School dropout potential scores are on a scale from 0 to 1.[12] Alcohol and cannabis use are on a scale from 1 to 7 (1=never, 2=just once, 3=less than monthly, 4=once a month, 5=1-2 times/week, 6=3+ times/week, 7=daily).[13] Cutoffs for severe or clinical outcomes were defined as follows: ≥90th percentiles for externalizing, internalizing, and bullying problems;[11] monthly use or more for alcohol and cannabis; and scores ≥ 0.40 for dropout potential[12]). ADHD indicates attention deficit and hyperactivity disorder symptoms.

eTable 2. Two-Group Model Estimates for Food Insecurity from Age 1.5 to 13 Years

Parameter	Estimate	Standard error	t	p-value
Low-risk trajectory of food insecurity				
Intercept	3.74	0.37	10.18	.00
Time, linear effects	0.96	1.19	0.81	.42
Time, quadratic effects	-0.52	1.06	-0.50	.62
High-risk trajectory of food insecurity				
Intercept	0.00	0.00	NA	NA
Time, linear effects	-1.14	1.10	-1.04	.30
Time, quadratic effects	0.46	1.00	0.46	.65

Data were compiled from the final master file of the Québec Longitudinal Study of Child Development (1998–2013), ©Gouvernement du Québec, Institut de la Statistique du Québec. Two-group trajectory modeling of the logit of probability of household food insecurity from 1.5 to 13 years of age (n=2032).

eTable 3. Prevalence of and Cumulative Exposure to Food Insecurity Between Ages 1.5–13 Years According to Group Trajectories

	**Low-risk trajectory of food insecurity**	**High-risk trajectory of food insecurity**
	N=1959	***N=73***
Food insecurity at 17 months, N (%):		
No	1854 (98.1%)	36 (52%)
Yes	35 (1.85%)	33 (48%)
Food insecurity at 4 years, N (%):		
No	1782 (98.7%)	19 (30%)
Yes	23 (1.27%)	44 (70%)
Food insecurity at 8 years, N (%):		
No	1214 (99.0%)	20 (51%)
Yes	12 (0.98%)	19 (49%)
Food insecurity at 10 years, N (%):		
No	1055 (98.9%)	9 (31%)
Yes	12 (1.12%)	20 (69%)
Food insecurity at 12 years, N (%):		
No	1184 (99.2%)	13 (33%)
Yes	9 (0.75%)	26 (67%)
Food insecurity at 13 years, N (%):		
No	1019 (98.9%)	16 (43%)
Yes	11 (1.07%)	21 (57%)
Count of food insecurity exposures, N (%):		
0	1857 (94.8%)	0 (0%)
1	102 (5.21%)	17 (23%)
2	0 (0.00%)	39 (53%)
3	0 (0.00%)	6 (8%)
4	0 (0.00%)	6 (8%)
5 or 6	0 (0.00%)	5 (7%)

Data were compiled from the final master file of the Québec Longitudinal Study of Child Development (1998–2013), ©Gouvernement du Québec, Institut de la Statistique du Québec.

eTable 4. Early Life Characteristics of Participants According to Attrition at Age 15

	Included	Excluded	p-value
	N=1441	***N=679***	
Child Characteristics			
Sex, N (%):			<.001
Female	752 (52.2%)	288 (42.4%)	
Male	689 (47.8%)	391 (57.6%)	
Internalizing behaviors at 29 months[a], Mean (SD)	1.15 (0.20)	1.15 (0.21)	.54
Externalizing behaviors at 29 months[b], Mean (SD)	1.50 (0.29)	1.51 (0.31)	.41
Family Characteristics			
Food insecurity trajectory, N (%):			.17
High risk	46 (3.19%)	27 (4.57%)	
Low risk	1395 (96.8%)	564 (95.4%)	
Socioeconomic status[c], Mean (SD)	0.10 (0.96)	-0.21 (1.04)	<.001
Single parent, N (%):			<.001
No	1344 (93.5%)	597 (88.6%)	
Yes	94 (6.54%)	77 (11.4%)	
Maternal age in years, Mean (SD)	29.0 (5.12)	28.6 (5.42)	.06
Mother born in Canada, N (%):			<.001
No	131 (9.10%)	122 (18.0%)	
Yes	1309 (90.9%)	556 (82.0%)	
Maternal depressive symptoms[d], Mean (SD)	1.34 (1.29)	1.52 (1.43)	.005

Data were compiled from the final master file of the Québec Longitudinal Study of Child Development (1998–2013), ©Gouvernement du Québec, Institut de la Statistique du Québec. Variables were measured when the child was 5 months of age, unless otherwise indicated.

[a]Assessed at 29 months; missing values were replaced with 17 months; 6 items from the Behavior Questionnaire[14] (e.g., is too fearful or anxious); score range: 0–8.

[b]Assessed at 29 months; missing values were replaced with 17 months; 10 items from the Behavior Questionnaire[14] (e.g., cannot sit still, is agitated); score range: 0–18.

[c]Standardized index based on annual gross income, parental education level and occupational prestige.[15]

[d]Assessed using a shortened version (12 items) of the Center for Epidemiologic Studies-Depression;[16] scores rescaled to 0–10.

eTable 5. Associations Between High-Risk Trajectory of Food Insecurity (1.5–13 years) and Mental Health and Functioning in Adolescence (15 years)

Outcome	Adjustment	β	95% CI	SE	t	p-value
Externalizing Problems						
ADHD	1	0.31	-0.01, 0.63	0.16	1.92	.06
	2	0.23	-0.1, 0.56	0.17	1.37	.17
	3	0.17	-0.16, 0.5	0.17	1.04	.30
Opposition	1	0.33	0.04, 0.63	0.15	2.24	.03
	2	0.20	-0.11, 0.5	0.15	1.27	.20
	3	0.15	-0.16, 0.45	0.15	0.94	.35
Conduct	1	0.49	0.2, 0.78	0.15	3.29	.001
	2	0.38	0.08, 0.68	0.15	2.48	.01
	3	0.32	0.02, 0.62	0.15	2.07	.04
Internalizing Problems						
Depression	1	0.22	-0.05, 0.5	0.14	1.58	.11
	2	0.24	-0.05, 0.53	0.15	1.63	.10
	3	0.18	-0.11, 0.47	0.15	1.24	.22
Social anxiety	1	0.12	-0.17, 0.4	0.15	0.79	.43
	2	0.11	-0.19, 0.41	0.15	0.72	.47
	3	0.10	-0.2, 0.4	0.15	0.63	.53
Generalized anxiety	1	0.08	-0.19, 0.36	0.14	0.59	.55
	2	0.10	-0.19, 0.39	0.15	0.68	.50
	3	0.05	-0.24, 0.33	0.15	0.33	.74
Substance Use						
Alcohol use	1	0.07	-0.23, 0.36	0.15	0.45	.65
	2	0.21	-0.1, 0.52	0.16	1.35	.18
	3	0.16	-0.14, 0.47	0.16	1.04	.30
Cannabis use	1	0.57	0.27, 0.86	0.15	3.82	<.001
	2	0.54	0.24, 0.85	0.15	3.53	<.001
	3	0.47	0.17, 0.76	0.15	3.06	.002
Social Adjustment Problems						
Peer bullying	1	0.50	0.21, 0.78	0.15	3.37	<.001
	2	0.44	0.14, 0.74	0.15	2.85	.004
	3	0.43	0.13, 0.73	0.15	2.79	.005
Dropout potential	1	0.75	0.47, 1.03	0.14	5.22	<.001
	2	0.40	0.12, 0.69	0.14	2.80	.005

© 2021 Paquin V et al. *JAMA Network Open*.

Outcome	Adjustment	β	95% CI	SE	t	p-value
	3	0.36	0.07, 0.64	0.14	2.47	.01

Data were compiled from the final master file of the Québec Longitudinal Study of Child Development (1998-2013), ©Gouvernement du Québec, Institut de la Statistique du Québec. Standardized coefficients (β) of linear regressions pooled over 40 multiply imputed datasets (n=1441). Models adjusted for (1) sex, (2) 1 + income sufficiency at 5 months, and (3) 2 + parental mental health (lifetime history of depression, measured at 29 months, and antisocial behaviors in adolescence, measured at 5 months). ADHD: attention deficit hyperactivity disorder symptoms. CI: confidence interval. SE: standard error.

eReferences

1. Côté SM, Orri M, Brendgen M, et al. Psychometric properties of the Mental Health and Social Inadaptation Assessment for Adolescents (MIA) in a population-based sample. *Int J Methods Psychiatr Res*. 2017;26(4). doi:10.1002/mpr.1566

2. Shrier I, Platt RW. Reducing bias through directed acyclic graphs. *BMC Med Res Methodol*. 2008;8(1):70. doi:10.1186/1471-2288-8-70

3. Barrett M. *Package "Ggdag": Analyze and Create Elegant Directed Acyclic Graphs*.; 2021. https://cran.r-project.org/web/packages/ggdag/ggdag.pdf

4. Suttorp MM, Siegerink B, Jager KJ, Zoccali C, Dekker FW. Graphical presentation of confounding in directed acyclic graphs. *Nephrol Dial Transplant*. 2015;30(9):1418-1423. doi:10.1093/ndt/gfu325

5. Shankar P, Chung R, Frank DA. Association of Food Insecurity with Children's Behavioral, Emotional, and Academic Outcomes: A Systematic Review. *J Dev Behav Pediatr*. 2017;38(2):16.

6. Davies C, Segre G, Estradé A, et al. Prenatal and perinatal risk and protective factors for psychosis: a systematic review and meta-analysis. *Lancet Psychiatry*. Published online March 2020:S2215036620300572. doi:10.1016/S2215-0366(20)30057-2

7. Jansen E, Lachman JM, Heinrichs N, Hutchings J, Baban A, Foran HM. Hunger in Vulnerable Families in Southeastern Europe: Associations With Mental Health and Violence. *Front Public Health*. 2020;8:115. doi:10.3389/fpubh.2020.00115

8. Helton JJ, Jackson DB, Boutwell BB, Vaughn MG. Household Food Insecurity and Parent-to-Child Aggression. *Child Maltreat*. 2019;24(2):213-221. doi:10.1177/1077559518819141

9. Leung CW, Stewart AL, Portela-Parra ET, Adler NE, Laraia BA, Epel ES. Understanding the Psychological Distress of Food Insecurity: A Qualitative Study of Children's Experiences and Related Coping Strategies. *J Acad Nutr Diet*. 2020;120(3):395-403. doi:10.1016/j.jand.2019.10.012

10. Kessler RC, McLaughlin KA, Green JG, et al. Childhood adversities and adult psychopathology in the WHO World Mental Health Surveys. *Br J Psychiatry*. 2010;197(5):378-385. doi:10.1192/bjp.bp.110.080499

11. Geoffroy M-C, Boivin M, Arseneault L, et al. Childhood trajectories of peer victimization and prediction of mental health outcomes in midadolescence: a longitudinal population-based study. *Can Med Assoc J*. 2018;190(2):E37-E43. doi:10.1503/cmaj.170219

12. Archambault I, Janosz M. Fidélité, validité discriminante et prédictive de l'indice de prédiction du décrochage. *Can J Behav Sci Rev Can Sci Comport*. 2009;41(3):187-191. doi:10.1037/a0015261

13. Landry M, Tremblay J, Guyon L, Bergeron J, Brunelle N. La Grille de dépistage de la consommation problématique d'alcool et de drogues chez les adolescents et les adolescentes (DEP-ADO) : développement et qualités psychométriques. *Drogue Santé Société*. 2004;3(1):20-37. doi:https://doi.org/10.7202/010517ar

14. Tremblay RE, Desmarais-Gervais L, Gagnon C, Charlebois P. The Preschool Behaviour Questionnaire: Stability of its Factor Structure Between Cultures, Sexes, Ages and Socioeconomic Classes. *Int J Behav Dev*. 1987;10(4):467-484. doi:10.1177/016502548701000406

15. Willms JD, Shields M. A measure of socioeconomic status for the National Longitudinal Study of Children. *Rep Prep Stat Can*. Published online 1996.

16. Roberts RE, Andrews JA, Lewinsohn PM, Hops H. Assessment of depression in adolescents using the Center for Epidemiologic Studies Depression Scale. *Psychol Assess J Consult Clin Psychol*. 1990;2(2):122-128. doi:10.1037/1040-3590.2.2.122

© 2021 Paquin V et al. *JAMA Network Open*.
